# A Combinatory Approach for Selecting Prognostic Genes in Microarray Studies of Tumour Survivals

**DOI:** 10.1155/2009/480486

**Published:** 2009-07-30

**Authors:** Qihua Tan, Mads Thomassen, Kirsten M. Jochumsen, Ole Mogensen, Kaare Christensen, Torben A. Kruse

**Affiliations:** ^1^Epidemiology, Institute of Public Health, University of Southern Denmark, J. B. Winsløws Vej 9B, 5000 Odense C, Denmark; ^2^Department of Biochemistry, Pharmacology and Genetics (BFG), Odense University Hospital, Sdr. Boulevard 29, 5000 Odense C, Denmark; ^3^Department of Obstetrics and Gynaecology, Odense University Hospital, Sdr. Boulevard 29, 5000 Odense C, Denmark

## Abstract

Different from significant gene expression analysis which looks for genes that are differentially regulated, feature selection in the microarray-based prognostic gene expression analysis aims at finding a subset of marker genes that are not only differentially expressed but also informative for prediction. Unfortunately feature selection in literature of microarray study is predominated by the simple heuristic univariate gene filter paradigm that selects differentially expressed genes according to their statistical significances. We introduce a combinatory feature selection strategy that integrates differential gene expression analysis with the Gram-Schmidt process to identify prognostic genes that are both statistically significant and highly informative for predicting tumour survival outcomes. Empirical application to leukemia and ovarian cancer survival data through-within- and cross-study validations shows that the feature space can be largely reduced while achieving improved testing performances.

## 1. Introduction

Similar to significant gene expression analysis, one demanding challenge in prognostic microarray experiments of tumour outcomes is the development of a powerful prognostic profile based on informative genes or features selected from a large pool of candidate genes measured on a relatively small number of arrays or tumour samples. Among the thousands of genes measured in an experiment, it is anticipated that only a limited number of genes are informative for prognostic purposes while a large number of genes are redundant or irrelative and thus can be ignored. Inclusion of uninformative genes for tumour outcome prediction only introduces unnecessary noise and will inevitably complicate model building and introduces computational difficulties. Obtaining a smaller subset of representative genes while retaining the prognostic characteristics of the original data should lead to a more accurate and efficient learning system with improved classification performance [1–3]. Furthermore, for prognostic purpose, predictive expression profiles built upon a limited number of genes are more useful in practice because their expression levels can be easily measured using economic and conventional methods, for example, the popular quantitative real-time PCR (qrt-PCR).

Different from significant gene expression analysis which looks for genes that are differentially regulated, feature selection in prognostic microarray studies aims at finding a subset of informative marker genes that are discriminative for prediction, ideally without redundancy. Ein-Dor et al. [4] reported that the set of outcome predictive genes is not unique due to the existence of multiple genes that are correlated with the clinical outcomes, and some of them may have only small differences in their correlations. Such a situation represents the hitting-set problem in finding the smallest set of features (hitting set) that encompass or characterize all the classes [5]. The difficulty in this context is the exponential search space created by all the possible genes or markers to be considered.

In the literature of prognostic microarray study, feature selection is predominated by the simple heuristic univariate gene filtering paradigm [6]. Since the univariate approach does not take into account the correlated or interactive structure among the genes, classifiers built upon genes so selected can be less accurate. More advanced approaches based on multivariate models have been considered, among them the variance-based dimension reduction [1, 7]. In prognostic microarray studies of tumour survival outcomes, the subject is further complicated by the censored observations and by the continuous nature of survival data for which simply engaging the gene selection and model building schemes for binary outcomes is inappropriate. In this paper, we introduce a combinatory feature selection strategy that integrates differential gene expression analysis for gene filtering using the supervised Cox regression model, unsupervised multivariate analysis for gene ranking, and redundancy reduction using the Gram-Schmidt process for identifying prognostic genes that are both statistically significant and highly informative for predicting tumour survivals. Empirical application of our method through both within- and cross-study validation shows that the feature space can be largely reduced while achieving improved testing performances.

## 2. Methods

### 2.1. Gene Filtering

We start with identifying genes that are differentially expressed in a microarray experiment by testing the marginal association between gene expression and survival time using the popular Cox regression model in which both censored and uncensored observations are used. In the Cox regression model, we assume that the hazard of death at time point *t* for subject *i* with the expression level of gene *j*, *x*_*i*,*j*_, is proportional to the baseline hazard of gene *j* at time *t*, *h*_*j*,0_(*t*), that is,

(1) $$h_{i}\left(t\right)=h_{j,0}\left(t\right)\exp\left(\beta_{j}x_{i,j}\right),$$

where *β*_*j*_ is the regression coefficient standing for the effect of gene *j* in affecting survival with a null hypothesis of *β*_*j*_ = 0. Our gene filtering can be performed according to the statistical significance level (*P*-value) or based on the number of genes to be retained. Note that the gene filtering step is done solely on the training set of the data.

### 2.2. Ranking of Significant Genes

Suppose there are *m* significant genes that survived the above gene filtering step in a microarray experiment with *N* samples in the training set, and we use *x*_*i*,*j*_ (*i* = 1, 2,…, *N*; *j* = 1, 2,…, *m*) to represent each expression levels for the genes in the feature space. Our objective here is to find a subset of informative marker genes or features of size *d* (*d* ≤ *m*) for predicting the outcomes of the testing samples. As mentioned above, the selected subset of genes should characterize the major features of the overall feature space of significant genes. Following Wei and Billings [8], we first calculate the squared-correlation coefficient for two vectors *x*_*s*_ and *x*_*t*_, *s*, *t* ∈ {1, 2,…, *m*}, each representing one feature in the feature space,

(2) $$r^{2}\left(x_{s},x_{t}\right)=\frac{{\left(x_{s}^{T}x_{t}\right)}^{2}}{\left(x_{s}^{T}x_{s}\right)\left(x_{t}^{T}x_{t}\right)}.$$

Equation (2) is done for all combinations of *s* and *t*. For each gene (e.g., *j*), we calculate the mean of the squared-correlation as *r*_mean_^2^(*j*) = (1/*n*)∑_*s* = 1_^*n*^ *r* ^2^(*x*_*s*_, *x*_*j*_). The gene with the highest mean is selected as the first most representative gene.

To select the second gene, each of the unselected genes indicated as *j* is orthogonalized to the selected gene using the Gram-Schmidt algorithm [8, 9] with the orthogonalization for the first selected gene *z*_1_ equaling to *x*_1_:

(3) $$z_{j}^{\left(2\right)}=x_{j}-\frac{x_{j}^{T}z_{1}}{z_{1}^{T}z_{1}}z.$$

Now we repeat the procedure in selecting the first gene by calculating the squared-correlation coefficient between each of the unselected genes *j* but using its orthogonalization and each of the *n* original genes and obtain its mean as *r*_mean_^2^(*j*) = (1/*n*)∑_*s* = 1_^*n*^ *r* ^2^(*x*_*s*_, *z*_*j*_^(2)^). The second gene is then selected as the one with the highest mean.

Likewise, in order to select the *k*th gene, each of the unselected genes *j* is orthogonalized to the *k*-1 selected genes as

(4) $$z_{j}^{\left(k\right)}=x_{j}-\frac{x_{j}^{T}z_{1}}{z_{1}^{T}z_{1}}z_{1}-\frac{x_{j}^{T}z_{2}}{z_{2}^{T}z_{2}}-\cdots -\frac{x_{j}^{T}z_{k-1}}{z_{k-1}^{T}z_{k-1}}z_{k-1}.$$

We again calculate the squared-correlation coefficient between each of the unselected genes *j* and each of the *n* original genes, and the mean for each of the genes, *r*_mean_^2^(*j*) = (1/*n*)∑_*s* = 1_^*n*^ *r* ^2^(*x*_*s*_, *z*_*j*_^(*k*)^). The *k*th gene is selected again as the gene with the highest mean. This process is repeated until all genes are selected and meanwhile ranked.

With the above procedure, a subset consisting of the most representative genes that accounts for the variation of the overall features with a high percentage can be selected. The data vector for each gene or feature can be approximated by a linear combination of the selected subset of features of size *d* (*d* ≤ *m*). Following Korenberg et al. [10], we can calculate the error reduction ratio (ERR) as a measurement for accounting for the variation in gene *j* by the *k*th gene (*k* = 1, 2,…, *d*) in the selected feature subset,

(5) $$\text{ERR}\left(j,k\right)=\frac{{\left(x_{j}^{T}z_{k}\right)}^{2}}{\left(x_{j}^{T}x_{j}\right)\left(z_{k}^{T}z_{k}\right)}\times 100\text{\%}.$$

The mean percentage of variation in the overall features or genes that are accounted for by gene *k* can be calculated as

(6) $$\bar{\text{ERR}}\left(k\right)=\left(\frac{1}{m}\right)\overset{m}{\underset{j=1}{\sum }}\text{ERR}\left(j,k\right).$$

Finally, the accumulated percentage of variation in the overall features or genes that are accounted for by the subset of *d* selected genes can be calculated as

(7) $$\bar{\text{SERR}}\left(d\right)=\overset{d}{\underset{k=1}{\sum }}\bar{\text{ERR}}\left(k\right).$$

$\bar{\text{SERR}}$ serves as a measurement in defining a subset of genes to sufficiently represent the overall features.

### 2.3. Optimization and Prediction Model Building

**Figure 1:**
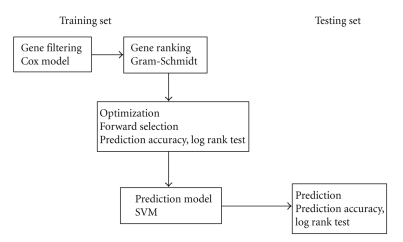
*Flow diagram of the combinatory procedure.* A combination of gene filtering using the supervised and gene ranking using the unsupervised analyses helps to assist the optimization step to identify a subset of prognostic genes for predicting the outcomes of an independent testing set.

After ranking significant genes, a subset of the most representative and informative marker genes are selected through optimization in the training set using forward selection which adds accumulatively each of the ranked genes (starting from the highest rank genes) to the prediction model and assesses model performance on the training set by calculating prediction accuracy (sensitivity, specificity) together with the chi-squared statistic for comparing differential survival between the predicted favourable and unfavourable groups using the log-rank test. The support vector machine (SVM) is used as the prediction model because of its popularity in machine learning. The simple linear kernel is chosen in model fitting. The free *R* package *e*1071 is used for fitting the SVM models. We set SVM probability of 0.5 as the cut-off (≥0.5 as favourable; <0.5 as unfavourable) in order to assign equal probability for the class membership. Sensitivity is calculated as the proportion of predicted favourable survivors in the long survivors (survival time > *mean* survival) and specificity as the proportion of predicted unfavourable survivors in those who died before the mean survival. In training the prediction models, the training set is divided into the observed favourable (survived over mean survival of the training set) and unfavourable (died before mean survival of training set) groups. Based on the class labels, classification models are trained based on different number of top rank genes and model performances evaluated. This optimization process results in the selection of a best performance model for the training set. The optimised model is then applied to the testing set. The whole process is illustrated in Figure 1.

## 3. Results

### 3.1. Adult Acute Myeloid Leukemia Data

**Figure 2:**
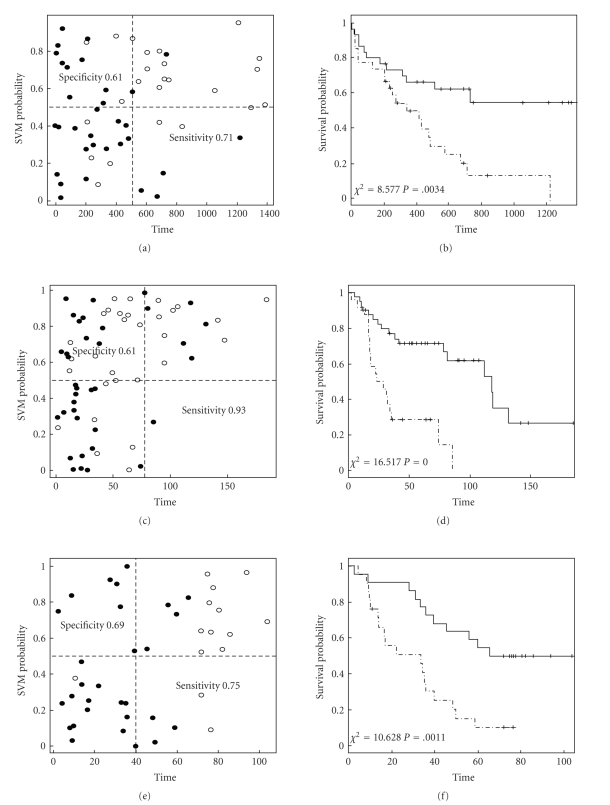
*Prediction results for within-study cross-validation analysis of three cancer data sets*. The results are shown as the SVM probability for each testing sample with censored observations in empty and uncensored in solid circles: (a), (c), (e) and as the Kaplan-Meier survival curves for the predicted favourable (solid) and unfavourable (dashed) groups: (b), (d), (f) with (a) and (b) for the adult acute myeloid leukemia data; (c) and (d) for the published ovarian cancer data; (e) and (f) for the in-house ovarian cancer data.

The above described procedure is first applied to a microarray data-set (containing 6.283 genes) from Bullinger et al. [11] who used gene expression profiling to identify subclasses of adult acute myeloid leukemia. An optimal 133-gene signature was developed which accurately predicted survival outcomes of an independent testing set (*P* = .006). Using exactly the same assignment of training (59 samples) and testing (57 samples) sets, we applied the above described procedure to the training set and selected a 23-gene expression signature for predicting the testing set (gene filtering with *P* < .005 leaving 70 highly significant genes for ranking and optimization; mean survival 384 days in the training set and 544 days in the testing set) with $\bar{\text{SERR}}(23)=0.82$. Figure 2 displays the prediction results for the testing set shown as the SVM probability for each testing sample with censored observations in empty and uncensored in solid circles (Figure 2(a), sensitivity 0.71 and specificity 0.61) and as the Kaplan-Meier survival curves for the predicted favourable (solid) and unfavourable (dashed) groups (Figure 2(b)). A log-rank test comparing differential survival between the two groups gave a chi-squared of 8.58 (df = 1) and a significance of *P* = .003.

### 3.2. Ovarian Cancer: Published Data

Bild et al. [12] reported oncogenic pathway deregulation identified by gene expression profiling in human cancers that can be used as a guide to targeted therapies. A relatively large collection of 132 ovarian tumour samples hybridized to the Affymetrix GeneChip Human Genome U133a Arrays each containing about 22,000 probe-sets were analysed as an example. Here we take the data and apply our method for selecting prognostic features to predict survival outcomes. We first randomly divided the data into training and testing sets (each containing 66 tumour samples). Following the steps in Figure 1, we filtered genes by picking up highly significant genes (*P* < .005, 194 genes) tested through fitting the Cox regression model to the training data. These genes were ranked and submitted to the optimization step in training the prediction models (mean survival in the training set 78 weeks and in testing set 64 weeks). A list of 32 top rank genes with $\bar{\text{SERR}}(32)=0.95$ was selected after optimization and used to build the final classifier for classifying the testing set. It can be seen from the result shown in Figure 2 that the model accurately classified long survivors into favourable and most of the short survivors into unfavourable groups with high probabilities (Figure 2(c), sensitivity 0.93 and specificity 0.61). As a result, the log-rank test for differential survival between the two groups gave a chi-squared as high as 16.52 (df = 1) which amounts to a *P*-value of 4.8e-05 (Figure 2(d)).

### 3.3. Ovarian Cancer: In-House Data

To show how the method can deal with data from small studies, we applied it to our in-house microarray data collected by Jochumsen et al. [13]. The data contains gene expression measurements for 43 ovarian tumour samples hybridized to the Affymetrix GeneChip Human Genome U133 Plus 2.0 Arrays, each containing about 55.000 probe-sets or genes. We introduce a leave-one-out (LOO) cross-validation for evaluating model performance considering the limited sample size. To do that, the above described procedure (from gene filtering to outcome prediction) is repeated every time a sample is left out for testing except that it is done for each given number of top rank genes accumulatively added beginning from the highest rank gene. The included subset of top rank genes that yield the biggest differential survival and highest prediction accuracy are selected. Every time a sample is dropped out, a new list of significant genes is identified using the remaining samples (the training set) from which the 100 top-most significant genes are ranked using the Gram-Schmidt process. Our method produced a 25-gene signature with $\bar{\text{SERR}}(25)=0.98$ that predicted the survival outcomes of all 43 samples (mean survival 39.8 months) with sensitivity 0.75 and specificity 0.69 (Figure 2(e)) and with highly significant differential survivals (chi-squared = 10.63 with df = 1, *P*-value = .0011) (Figure 2(f)).

### 3.4. Ovarian Cancer: Cross-Study Validation

**Figure 3:**
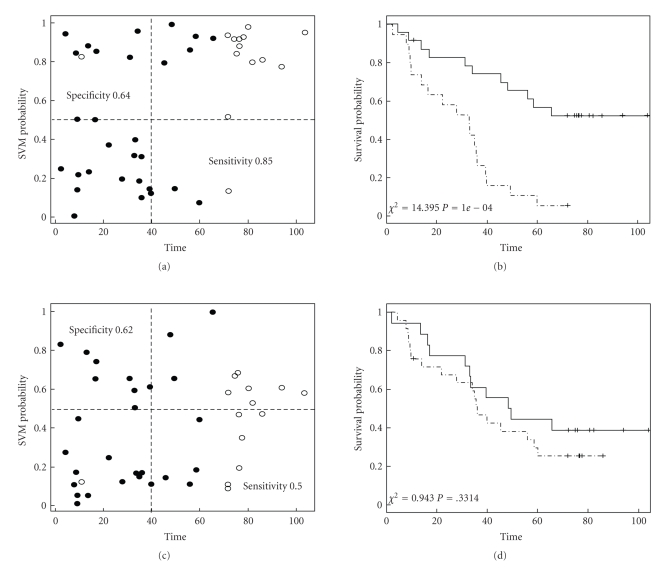
*Prediction results for cross-study validation analysis of the two ovarian cancer data sets.* The results are shown as the LOO SVM probability for each sample in the in-house data with censored observations in empty and uncensored in solid circles (a) and as the Kaplan-Meier survival curves for the predicted favourable (solid) and unfavourable (dashed) groups (b). Results from analysis using genes ranked by their statistical significances are shown in (c) and (d).

As the above two data sets are all on ovarian cancer, we additionally conducted a cross-study validation to show performance of the features selected using our method and compare it with that from genes selected only according to their statistical significances. The analysis also takes advantage of the same platform of microarrays used in the two studies, that is, the Affymetrix GeneChip Human Genome U133 Arrays although with different versions. Since the array for our in-house data (U133 plus 2.0, 55.000 probe-sets) is inclusive of the published array (U133a, 22.000 probe-sets), cross-study validation is only possible for validating genes selected from the published array data. Note here we used the whole published data set of 132 tumour samples for gene filtering (*P* < .001, 277 genes survived), ranking, and optimization. A 31-gene signature was established with $\bar{\text{SERR}}(31)=0.91$. The subset of predictive genes was then submitted to our in-house data for validation using LOO. In Figure 3, we show the LOO cross-validation probability plotted against observation time for each of the 43 samples (Figure 3(a)) and the Kaplan-Meier survival curves for the predicted good (solid) and poor (dashed) outcome groups (Figure 3(b)). As one can see, the classifier based on the 31-gene signature separates nicely the long survivors from most of the short survivors (Figure 3(a), sensitivity = 0.85 and specificity = 0.64). The log-rank test for differential survival between the two groups gave a chi-squared of 14.40 (df = 1) and a *P*-value of 1e-04 (3b). As a comparison, we also performed cross-study validation on genes selected through optimization on genes ranked according to their statistical significances. A subset of 45 genes was selected after optimization on the published data and applied to our in-house data for LOO validation. Unfortunately the prediction model failed to predict survival outcomes of our in-house samples (sensitivity = 0.62 and specificity = 0.5; chi-squared = 0.94 with df = 1 and *P*-value = .33).

## 4. Discussion

We have shown that our combinatory approach can be used for selecting statistically significant and highly informative genes for predicting tumour survival outcomes in microarray studies. The method removes redundant genes that, although statistically significant, have low impact on prediction so that improved prediction on an independent testing set is expected. Our results indicate that “significant” features selected using the genewise approaches can contain irrelative or redundant genes that serve only to complicate model building for a classifier. Our empirical result helps to further emphasize the difference between significant and prognostic gene expression analyses because the former only looks for genes statistically significantly regulated (including correlated genes coexpressed in a biological pathway) while the latter, on the other hand, tries to extract prognostic genes that are not only statistically significant but also highly informative in characterizing tumour outcomes.

Our combinatory approach consists of both the supervised univeriate differential gene expression analysis for gene filtering and the unsupervised multivariate algorithm for ranking the significant genes in a consecutive manner. The ranking of genes assists subsequently the forward optimization step in determining the final subset of informative genes for building up the final classifier. Given the large number of genes measured in an experiment, it is important to ensure that statistically highly significant genes are picked up after gene filtering in order to form a meaningful candidate feature space for subsequent ranking and optimization. This is necessary because ( 1) the gene ranking step works only if the candidate feature-space contains genes that are highly correlated with tumour survival outcomes although some of them may be of only minor impact in prediction; ( 2) picking up highly significant genes helps to reduce the number of false positive genes that are included in the candidate feature space; and ( 3) a good candidate feature space can help to increase computational efficiency because the computation load goes up exponentially with the number of genes in the feature space.

Feature redundancy reduction not only helps to improve performance and generalization of a classifier, it is also advantageous for clinical applications. With the confirmed subset of highly prognostic genes, routine bioinstrumentations for gene expression level measurement such as the qrt-PCR [14] can be used to reduce the cost of prognostic gene expression analysis in clinical applications.

It is necessary to mention that the survival analysis in the gene filtering step makes full use of both censored and uncensored samples in identifying differentially expressed genes. The gene filtering step using survival analysis model can be generalized to binary or categorical clinical outcomes such as tumour metastasis status where corresponding statistical models (e.g., *t*-test, ANOVA etc.) can be introduced for determining significantly regulated genes. The only difference is that, in training the classifier, the two-step procedure is reduced to one-step because classes of the training samples are already well defined.
